# Determinants of Neurological Outcome Following Elective and Emergency Open Thoracoabdominal Aortic Aneurysm Repair—A Retrospective Multi-Center Study

**DOI:** 10.3390/jcm13185473

**Published:** 2024-09-14

**Authors:** Jelle Frankort, Panagiotis Doukas, Christian Uhl, Nelly Otte, Julia Krabbe, Barend Mees, Michael J. Jacobs, Alexander Gombert

**Affiliations:** 1 Department of Vascular Surgery, Medical Faculty, RWTH Aachen University, 52074 Aachen, Germanym.jacobs@mumc.nl (M.J.J.);; 2 Department of Vascular Surgery, MUMC+ Maastricht, 6229 HX Maastricht, The Netherlands; barend.mees@mumc.nl; 3 Institute of Occupational, Social and Environmental Medicine, Medical Faculty, RWTH Aachen University, 52074 Aachen, Germany

**Keywords:** thoracoabdominal aortic aneurysms, open surgical repair, neurological complications

## Abstract

**Background/Objectives**: This study aimed to evaluate and establish the incidence of all types of neurological complications at our high-volume reference center for open TAAA repair in the Netherlands and Germany. Additionally, we sought to identify predictors for various neurological complications. **Methods**: This retrospective study was conducted in accordance with the STROBE guidelines, with the aim of reporting neurological outcomes for all patients who underwent open thoracoabdominal aortic aneurysm repair at two centers (Maastricht-Aachen) from 2000 to 2023, and to examine the association between these outcomes and pre- and perioperative parameters. The primary endpoints of the study were all-cause mortality, spinal cord ischemia (SCI), stroke, intracerebral bleeding (ICB), critical illness polyneuropathy/myopathy (CIP/CIM), and recurrent laryngeal nerve paralysis. **Results**: A total of 577 patients were operated on for open TAAA repair in two centers. The total in-hospital mortality rate was 20.6%, while the elective cases in-hospital mortality rate was 14.6%. In all, 28.2% of patients experienced neurological complications. The spinal cord ischemia rate was 7.5%, intracerebral bleeding 3.6%, stroke 5.9%, critical illness polyneuropathy 3.5%, and laryngeal nerve paresis 5.7%. Crawford extent II was significantly associated with increased neurological complications (OR 2.05, 95% CI 1.39–3.03, *p* = 0.003), while Crawford extent III and IV were significantly associated with fewer postoperative neurological complications (OR 0.61 (0.38–0.98) *p* = 0.04) (OR 0.52 (0.30–0.92) *p* = 0.02). Preoperative ASA score > 3 (OR 1.76, 95% CI 1.16–2.67, *p* = 0.007), COPD (OR 1.82, 95% CI 1.19–2.78, *p* = 0.006), massive intraoperative transfusion (OR 1.48, 95% CI 1.01–2.17, *p* = 0.04), and reinterventions during hospital stay (OR 1.98, 95% CI 1.36–2.89, *p* < 0.001) and surgery time (*p* =< 0.001) were significantly associated with neurological complications. Patients with neurological complications had higher rates of other postoperative morbidities. **Conclusions**: Neurological complications after open TAAA repair remain a significant concern, with identified risk factors associated with increased morbidity, mortality, and resource utilization. Identifying at-risk patients could potentially lead to a reduction in neurological complications.

## 1. Introduction

Managing thoraco-abdominal aortic aneurysms (TAAAs) presents considerable challenges and open repair stands as a fundamental approach for both elective and emergency cases, particularly when endovascular repair proves not suitable, such as those afflicted with connective tissue diseases or aortic infections [1,2,3]. Nevertheless, the invasive nature of open TAAA repair introduces a notable incidence of morbidity and mortality [4]. Among the all the complications, neurological complications stand out as devastating for both patients and surgeons. These complications include spinal cord ischemia (SCI), stroke, intracerebral bleeding (ICB), and critical illness neuropathy (CIP). Addressing and mitigating these neurological challenges are crucial aspects of managing patients after open TAAA repair. To mitigate these risks, perioperative measures such as staged repair of the aneurysm, cerebrospinal fluid drainage, intraoperative neuromonitoring, and permissive hypertension are applied [5,6]. Nevertheless, neurological complications are not uncommon after open TAAA repair. A recent systematic review found a pooled SCI rate of 7.6% (96% CI, 6.2–9.3) for open TAAA repair, but there is wide range among different studies [7]. Research on neurological complications following open thoracoabdominal aortic aneurysm (TAAA) repair has predominantly focused on spinal cord ischemia (SCI) and stroke rates. However, studies examining other types of neurological complications, such as intracerebral bleeding (ICB) or critical illness polyneuropathy (CIP), are scarce or nonexistent in the current literature. Moreover, minor neurological complications like recurrent laryngeal nerve paralysis have been underreported or not previously addressed in the context of TAAA repair. This study aimed to evaluate and establish the incidence of all types of neurological complications at our high-volume reference center for open TAAA repair in the Netherlands and Germany. Additionally, we sought to identify predictors for various neurological complications.

## 2. Materials and Methods

In this study, we retrospectively reviewed the medical records of 577 patients who underwent open TAAA repair in two centers between 2006 and 2023. The study was reviewed and approved by the ethics committee of the University Hospital RWTH Aachen (EK004/14, approval date: 21 May 2019) and was designed according to the STROBE criteria and the Declaration of Helsinki [8].

Inclusion criteria comprised elective or emergency open repair for thoracoabdominal aortic aneurysm (TAAA), categorized by the Crawford classification [9]. Emergency or urgent repair was defined as treatment within 24 h because of symptomatic TAAA without signs of open rupture, namely severe back or abdominal pain with concomitant TAAA > 50 mm and absence of further plausible explanation for the symptoms. A clear separation between a covered rupture and a symptomatic TAAA was not possible in every case [4]. Mycotic TAAA were excluded. The definitive treatment plan was collaboratively determined by a multidisciplinary team involving vascular surgeons, cardiac surgeons, interventional radiologists, and anesthetists. Endovascular options were not considered viable at the judgment time due to morphological reasons. Patients with connective tissue disease-related TAAA were primarily managed through open repair and were included in the study.

### 2.1. Surgery

The surgical protocol for open thoracoabdominal aortic aneurysm (TAAA) repair in our center has been documented before [4,10]. The protocol encompassed double lumen tube intubation with cerebrospinal fluid drainage (CSFD), perioperative monitoring of motor evoked potentials (MEPs) if possible, and positioning the patient on a beanbag in a modified right lateral decubitus posture. The operating table was elongated to facilitate optimal access to the thoracic cavity. The surgical approach involved sequential aortic clamping when feasible, femorofemoral extracorporeal circulation (ECC) with distal aortic perfusion, selective visceral perfusion, and mild hypothermia (32–33 °C). Intraoperatively, if there are any changes in neuromonitoring signals, the surgical team is immediately notified. Significant change or loss of MEP signals can indicate impending or actual spinal cord ischemia, prompting immediate intervention through permissive hypertension, increase cerebrospinal fluid drainage or intercostal artery reimplantation. In the case of stroke, however, definitive diagnosis during surgery is not possible. Therefore, we complete the planned procedure and immediately perform a postoperative CT angiography of the head. Subsequently, consultation with neurology and neuroradiology departments takes place. Based on imaging findings and clinical presentation of the patient, we determine the most appropriate intervention, which may include conservative management or emergency intracranial thrombectomy. Custodiol (Bensheim, Germany) has been employed for renal perfusion instead of blood perfusion since 2014. Depending on the aneurysm’s extent, surgical access via thoracolaparotomy through the sixth to eighth intercostal space was adopted, and aortic reconstruction proceeded from proximal to distal and in cases of dissection from distal to proximal [4,10].

### 2.2. Definitions and Protocol

Significant neurological complications were characterized by occurrences such as stroke, cerebral bleeding, spinal cord ischemia (SCI), or critical illness polyneuropathy following TAAA surgery [11,12,13]. SCI, specifically, was identified as bilateral lower extremity motor weakness and sensory loss to pain and temperature modalities during the peri- or postoperative period, with the preservation of vibration and position sense [14,15]. Clinical assessments for neurological dysfunction were conducted by a physician during the intensive care stay, with a frequency of every four to six hours, or more frequently if necessary. Critical illness polyneuropathy, characterized by severe muscle weakness and the inability to be weaned from the ventilator, was also part of the defined complications [16]. Stroke or ICB were diagnosed through computed tomographic imaging [13,17]. In case of neurological complications, the discharge plan was tailored to the severity and nature of each patient’s neurological deficit. The majority of patients with significant neurological impairments were transferred to specialized rehabilitation centers equipped to manage complex neurological conditions. These facilities offer intensive, multidisciplinary rehabilitation programs designed for patients recovering from spinal cord injuries, strokes, or other neurological complications. For patients with milder neurological deficits, discharge to home with outpatient rehabilitation services was sometimes deemed appropriate. This decision was made on a case-by-case basis, considering factors such as the patient’s functional status, home support system, and access to outpatient neurological care.

### 2.3. Statistics

Data analysis was performed using SPSS 26 (Statistical Package for the Social Sciences, Inc., Chicago, IL, USA). All data are presented as number of patients (n) and their relative amount (%) or median with interquartile range (IQR). For events occurring in at least three patients, odds ratios (ORs) and corresponding 95% confidence intervals (CI) were determined for estimate of magnitude of association. For comparison between two groups, Mann–Whitney U-test or Chi^2^ test was used. For identification of predictors of neurological complication as outcome, a binary logistic regression was performed. With neurological complication as dependent variable, independent variables were included that showed significant differences in patients demographics or perioperative characteristics and resulted in the best fit (exclusion of ASA ≥ 3). For evaluation of fit, Nagelkerke’s R^2^ and Hosmer–Lemeshow were performed.

Analysis differences were assumed to be significant with *p* < 0.05.

## 3. Results

This retrospective multi-center study includes 577 patients who underwent open TAAA repair between 2000 and 2023 at centers in Maastricht, Netherlands and Aachen, Germany. Of these patients, 400 (69%) were male, with a median age of 61 years (ranging from 14 to 83 years). The distribution of patients by Crawford classification was as follows: type I, 29.6%; type II, 27.7%; type III, 21.5%; type IV, 15.9%; and type V, 5.0%. One hundred and two patients (17.7%) underwent emergency or urgent procedures. In all, 28% of patients had a history of aortic surgery, and 19.6% had connective tissue disease. Preoperative ASA score > 3 (OR 1.76, 95% CI 1.16–2.67, *p* = 0.007) and chronic obstructive pulmonary disease (COPD) (OR 1.82, 95% CI 1.19–2.78, *p* = 0.006) were found to be significantly associated with postoperative neurological complications. (Table 1) Acute kidney injury (AKI) occurred in 242 of the 577 patients (41.9%), which at least had a 2-fold increase in their preoperative creatinine levels. Of these, 131 patients (22.7% of the total cohort) had more than a 3-fold increase in their preoperative creatinine levels. A total of 33 patients (5.7%) required permanent renal replacement therapy.

The total in-hospital mortality rate was 20.6%, while the elective cases in-hospital mortality rate was 14.6%. A substantial proportion of patients, 28.2%, experienced neurological complications. (Table 2) The SCI rate was 7.5% (43/577), and the ICB rate was 3.6% (21/577). Stroke was identified in 5.9% (34/577) of patients, while the CIP/CIM rate was 3.5% (20/577). Approximately 5.7% of patients (33/577) experienced laryngeal nerve paresis (Table 2).

Crawford extent II was found to be significantly associated with postoperative neurological complications (OR 2.05 (1.39–3.03) *p* = 0.003), while Crawford extent III and IV were significantly associated with fewer postoperative neurological complications (OR 0.61 (0.38–0.98) *p* = 0.04) (OR 0.52 (0.30–0.92) *p* = 0.02). Furthermore, massive intraoperative transfusion was also found to be a significant factor for neurological complications (OR 1.48 (1.01–2.17) *p* = 0.04). Additionally, patients who underwent reinterventions during their hospital stay had significantly more neurological complications (OR 1.98 (1.36–2.89) *p* =< 0.001) (Table 3).

Binary logistic regression for neurological complication as dependent variable demonstrated a weak relationship between the predictors and the outcome (Nagelkerke’s R^2^ < 0.2). In the model COPD, Crawford II and surgery time could be identified as significant predictors. With COPD or Crawford II, the risk for a neurological complication would be 1.6 to roughly 2-fold higher than without. For every minute that surgery time is increased, the risk for a neurological complication would increase by 0.2% (Table 4).

Patients with neurological complications exhibited a significantly higher incidence of tracheotomies (OR, 3.38; 95% CI, 2.06–5.53; *p* < 0.001), pneumonia (OR, 2.67; 95% CI, 1.84–3.88; *p* < 0.001), sepsis (OR, 2.49; 95% CI, 1.68–3.68; *p* =< 0.001), acute respiratory distress syndrome (ARDS) (OR 1.88; 95% CI, 1.04–3.40; *p* = 0.03), wound complications (OR, 1.77; 95% CI, 1.11–2.81; *p* = 0.02), and permanent dialysis (OR, 3.43; 95% CI, 1.79–6.58; *p* < 0.001) (Table 5).

A lengthier surgery duration was found to be significantly associated with neurological complications (*p* =< 0.001). Patients with neurological complications experienced a more extended hospital stay (*p* < 0.001), were mechanically ventilated for a longer period (*p* < 0.001), and spent more time in the intensive care unit (*p* < 0.001) (Table 6).

## 4. Discussion

Open surgical repair of TAAA is associated with relevant morbidity and mortality [14,18]. Our study reveals that neurological complications remain a significant concern in open thoracoabdominal aortic aneurysm (TAAA) repair, with an overall incidence of 28.2%. In particular, we found spinal cord ischemia (SCI) rates of 7.5%, stroke rates of 5.9%, and intracerebral bleeding (ICB) rates of 3.6%. Notably, we report for the first time on critical illness polyneuropathy/myopathy (CIP/CIM) rates (3.5%) and recurrent laryngeal nerve paralysis (5.7%). Risk factors include Crawford extent II aneurysms, preoperative ASA score > 3, COPD, massive intraoperative transfusion, and reinterventions during hospital stay. These complications were associated with increased morbidity, longer hospital stays, and higher resource utilization. Neurological complication rates, even after implementing multiple strategies ranging from permissive hypertension, neuromonitoring, to cerebrospinal fluid drainage to prevent them, are still relatively high [5,10,19]. Our study shows a SCI rate of 7.5%, which is in line with other big reference centers worldwide, were SCI rates range from 5.7% to 13.70% [18,20,21]. This consistency across centers highlights the persistent challenge in preventing SCI, even with advanced techniques. A meta-analysis by Gaudino et al. found a pooled permanent SCI rate of 4.7% (95% CI, 3.9–5.6%) for TAAA repair [7]. The small variation in reported rates may be due to differences in patient populations, surgical techniques, and definitions of SCI across studies. We found a stroke rate of 5.9%, which is also in line compared to other centers, who published there results, where the stroke rate ranged from 2% to 8.4 [22,23,24].

Nevertheless, the primary emphasis of other studies regarding neurological complications rests on SCI rates and stroke rates, while other neurological complications are insufficiently represented and inadequately reported. Moreover, the data on intracerebral bleeding are meager. Coselli et al. mentions a low ICB rate of 0.9% [18]. The generalizability of these findings relative to our ICB rate of 3.6% is challenging due to the limited quantity of other studies reporting ICB. Our study is the first to report on CIP/CIM after open TAAA repair. We found a CIP/CIM rate of 3.5% and a recurrent laryngeal nerve paralysis rate of 5.7%, both of which can significantly impact patient recovery and quality of life. However, it is not feasible to compare these outcomes with other studies due to the scarcity of publications on this subject.

Preoperative risk stratification is crucial, as we found that factors such as aneurysm extent and preexisting comorbidities can significantly influence neurological outcomes. Additionally, older patients present with higher rates of adverse outcomes, emphasizing the need for careful consideration of the risks and benefits in this population [25]. Intraoperatively, strategies such as sequential aortic clamping, reimplantation of intercostal arteries, and maintenance of adequate spinal cord perfusion pressure are essential [26].

Notably, we found that Crawford extent II TAAA was significantly associated with increased risk of neurological complications (OR 1.99, 95% CI 1.36–2.95, *p* = 0.004). This is consistent with the understanding that more extensive aneurysms involving a larger portion of the aorta carry higher risks of spinal cord ischemia and other neurological complications. Conversely, Crawford extent IV TAAA was associated with fewer neurological complications (OR 0.51, 95% CI 0.29–0.90, *p* = 0.02), likely due to the more limited extent of the repair and the fact that most of the important intercostal arteries are located in the thoracic aorta [27,28].

The strong association between neurological complications and other postoperative morbidities, including tracheostomies, pneumonia, sepsis, and permanent dialysis, underscores the influence of these events on patient recovery and long-term consequences. These complications also result in extended hospital stays for patients, which increase costs and burden. This highlights the necessity for thorough perioperative care, and most definitely ICU care, as well as constant monitoring to detect and manage complications promptly in the patients at risk [23].

The regression analysis revealed significant differences between the groups with and without neurological complications. Comparing our predictors to other studies is difficult due the lack of data. A study from Coselli et al. also found extent II TAAA repair, among coronary artery disease and chronic symptoms, as an independent predictor for spinal cord ischemia [18]. However, the characteristics that differed between these groups could not be collectively used as reliable predictors to estimate which patients are at risk for neurological complications or to quantify that risk. This suggests a complex model where relevant factors that significantly determine increased risk have yet to be fully identified. These unidentified factors may include anatomical variations, genetic predispositions, or even quality-related aspects such as surgeon experience, anesthesia management, and intensive care unit practices. The multifaceted nature of neurological complications following TAAA repair likely involves intricate interactions between patient-specific factors and perioperative variables that our current model does not fully capture. Our findings underscore the need for further studies to examine these potential factors.

Comparing the outcomes of open TAAA repair with those of endovascular TAAA repair is difficult due to differences in baseline characteristics and patient demographics, as open TAAA repair is typically reserved for a limited subset of patients who have unfavorable anatomy for endovascular TAAA repair or have a genetically triggered aortopathy. A systematic review conducted by Ellahi et al. investigating open TAAA and endovascular TAAA repair showed no significant differences in stroke or paraplegia rates between the two [29].

A limitation of this study is its retrospective nature, hampering the reliance on available medical records. Ideally, a prospective study with a standardized follow-up protocol would provide more evidence. In addition, this study did not evaluate the impact of changes specific for indicating open TAAA repair or advances in perioperative care; for instance, we were unable to definitively assess the impact of different renal perfusion methods on neurological outcomes in this study. During the transition period from blood perfusion to Custodiol, our documentation was not consistently detailed enough to accurately determine which method was used for each patient.

Another limitation of this study is the potential for confounding factors and the complex interplay between neurological and other postoperative complications. The causal relationship between neurological deficits and other adverse outcomes remains unclear. Neurological complications may predispose patients to additional morbidities such as prolonged mechanical ventilation, pneumonia, or renal failure, as found in our study. Conversely, non-neurological complications like hemodynamic instability or systemic inflammatory responses could exacerbate or precipitate neurological deficits [30]. This bidirectional relationship makes it challenging to establish clear causality. Furthermore, shared risk factors like extensive aneurysm repair, prolonged operative time, or preexisting comorbidities may independently increase the likelihood of both neurological and non-neurological complications. Studies with detailed temporal analyses and multivariate models are needed to better elucidate these complex associations and potential causal pathways [31,32].

## 5. Conclusions

Neurological complications after open thoracoabdominal aortic aneurysm repair remain a significant concern, with risk factors including aneurysm extent, ASA score, COPD, massive intraoperative transfusion, and reinterventions during hospital stay. These complications are associated with increased morbidity and mortality. Identifying the patients at risks could potentially lead to a reduction in neurological complications.

## Figures and Tables

**Table 1:** Patient demographics: comparison of patients with and without neurological complications.

	Overall	Neurological Complication	Without Neurological Complication	OR (95% CI)	*p*-Value ^#^
	Median (IQR)	Median (IQR)	Median (IQR)		
Age, years	61 (504–68)	59 (494–66)	62 (51–69)	-	0.14
BMI (kg m^−2^)	25.2 (22.5–28.5.0)	25.1 (22.6–28.4.8)	25.2 (22.4–28.3)	-	0.50
	n	n (%)	n (%)		
Male	400	121 (74.7)	279 (68.0)	1.30 (0.86–1.96)	0.12
Smoking	293	82 (63.6)	211 (67.0)	0.86 (0.56–1.32)	0.49
Obesity (BMI ≥ 30)	77	27 (17.4)	50 (13.6)	1.35 (0.81–2.24)	0.25
ASA ≥ 3	315	100 (70.4)	215 (57.5)	1.76 (1.16–2.67)	0.007 *
Diabetes mellitus	43	11 (6.7)	32 (7.7)	0.86 (0.42–1.75)	0.68
Renal failure	238	78 (49.1)	160 (40.0)	1.44 (1.00–2.09)	0.05
Hypertension	448	135 (83.9)	313 (77.1)	1.54 (0.96–2.49)	0.08
Congestive heart failure	76	22 (23.4)	54 (29.3)	0.74 (0.41–1.31)	0.29
Coronary heart disease	126	41 (30.8)	85 (28.8)	1.01 (0.70–1.72)	0.67
COPD	119	46 (28.8)	73 (18.2)	1.82 (1.19–2.78)	0.006 *
Myocardial infarction	71	17 (12.7)	54 (18.8)	0.63 (0.35–1.13)	0.12
Stroke	30	10 (9.4)	20 (10.3)	0.91 (0.41–2.01)	0.81
PAD	62	16 (12.9)	33 (12.6)	1.03 (0.54–1.95)	0.93
PCI	100	36 (34.6)	64 (30.9)	1.18 (0.72–1.95)	0.51
CABG	41	15 (11.3)	26 (8.9)	1.30 (0.66–2.54)	0.45
Previous aortic surgery	162	63 (59.4)	99 (50.3)	1.45 (0.90–2.34)	0.13

^#^ For age and BMI comparison via Mann–Whitney U-test, otherwise Chi^2^-test. * *p* =< 0.05. ASA: American Society of Anesthesiologists; BMI: Body Mass Index; CABG: Coronary Artery Bypass Grafting; CI: Confidence Interval; COPD: Chronic Obstructive Pulmonary Disease; OR: Odds Ratio; PAD: Peripheral Artery Disease; PCI: Percutaneous Coronary Intervention.

**Table 2:** Patient demographics: neurological complications in detail.

Neurological Complication	n (%)
Any neurological complication	163 (28.2%)
Spinal cord deficit	43 (7.5%)
Stroke	34 (5.9%)
ICB	21 (3.6%)
Encephalopathy	2 (0.3%)
Epileptic seizure	3 (0.5%)
Vocal cord paresis	33 (5.7%)
CIP/CIM	20 (3.5%)
Other sensitivity disorders	7 (1.2%)

**Table 3:** Perioperative comparison of patients with and without neurological complications.

	Overall n	Neurological Complication n (%)	Without Neurological Complication n (%)	OR (95% CI)	*p*-Value ^#^
Rupture	58	18 (11.1)	40 (9.7)	1.16 (0.64–2.09)	0.62
Urgent or emergency repair	102	27 (16.6)	75 (18.2)	0.89 (0.55–1.45)	0.65
Crawford classification					
Crawford I	171	51 (45.5)	120 (41.0)	0.73 (0.49–1.09)	0.60
Crawford II	160	63 (63.0)	97 (30.7)	2.05 (1.39–3.03)	0.003 *
Crawford III	124	26 (19.0)	98 (31.1)	0.61 (0.38–0.98)	0.04 *
Crawford IV	92	17 (11.6)	75 (22.2)	0.52 (0.30–0.92)	0.02 *
Crawford V	29	6 (3.8)	23 (1.0)	0.74 (0.30–1.86)	0.35
Intercostal artery implantation	225	73 (45.1)	152 (37.0)	1.40 (0.97–2.02)	0.08
Massive transfusion	281	90 (59.6)	191 (49.9)	1.48 (1.01–2.17)	0.04 *
Incidental splenectomy	65	19 (10.9)	46 (11.2)	1.05 (0.60–1.86)	0.86
Arrythmia	26	7 (68.6)	19 (10.9)	0.60 (0.24–1.48)	0.27

^#^ Chi^2^-test. * *p* =< 0.05.

**Table 4:** Binary logistic regression with best fit for univariately significant variables as independent variables. Prediction of neurological complication as dependent variable.

Model	Chi2	df	*p*-Value	Pseudo R^2^ (Nagelkerke)	Hosmer–Lemeshow
	33.72	6	<0.001 **	0.10	0.92
**Independent variables**	**B**	**SE**	**Wald**	**OR (95% CI)**	***p*** **-value**
Sex	−0.30	0.24	1.56	0.74 (0.46–1.19)	0.21
Age	−0.01	0.01	2.60	0.99 (0.97–1.00)	0.11
COPD	0.67	0.24	7.48	**1.95 (1.21–3.14)**	0.006 *
Crawford II	0.47	0.24	3.91	**1.60 (1.00–2.54)**	0.048 *
Mass transfusion	−0.04	0.23	0.04	0.96 (0.61–1.50)	0.85
Surgery time, incision-suture, minutes	0.002	0.001	4.99	**1.002 (1.000–1.004)**	0.03 *

COPD: Chronic Obstructive Pulmonary Disease; * *p* =< 0.05. ** *p* =< 0.001. SE: Standard Error; Morbidity: comparison of patients with and without neurological complications for various complications.

**Table 5:** Morbidity: comparison of patients with and without neurological complications for various complications.

	Overall n	Neurological Complication n (%)	Without Neurological Complication n (%)	OR (95% CI)	*p*-Value ^#^
Tracheotomy	109	59 (53.6)	50 (25.5)	**3.38 (2.06–5.53)**	<0.001 *
Pneumonia	247	98 (60.1)	149 (36.1)	**2.67 (1.84–3.88)**	<0.001 *
Urinary infection	46	11 (13.7)	35 (8.5)	0.78 (0.38–1.57)	0.48
Sepsis	152	65 (39.9)	87 (21.1)	**2.49 (1.68–3.68)**	<0.001 *
Complication with graft	39	18 (17.5)	21 (11.4)	1.64 (0.83–3.25)	0.15
Myocardial infarction	18	6 (3.7)	12 (2.9)	1.28 (0.47–3.48)	0.63
ARDS	51	21 (12.9)	30 (7.3)	**1.88 (1.04–3.40)**	0.03 *
Wound complication	93	36 (22.2)	57 (13.9)	**1.77 (1.11–2.81)**	0.02 *
Permanent renal replacement	40	22 (13.7)	18 (4.4)	**3.43 (1.79–6.58)**	<0.001 *
Reintervention	186	71 (43.6)	115 (28.0)	**1.98 (1.36–2.89)**	<0.001 *

^#^ Chi^2^-test, * *p* =< 0.05. ARDS: Acute Respiratory Distress Syndrome.

**Table 6:** Comparison of patients with and without neurological complications for surgery time, hospital stay, and ventilation/weaning.

	Overall	Neurological Complication	Without Neurological Complication	*p*-Value ^#^
	n	Median (IQR)	Median (IQR)	
Surgery time, incision-suture, minutes	544	427 (330–496)	369 (298–449)	<0.001 *
Duration of hospital stay, days	570	32 (19–49)	22 (15–32)	<0.001 *
Duration of mechanical ventilation, hours	246	43 (6–528)	6 (3–35)	<0.001 *
Intensive care and weaning, days	521	17 (6–32)	8 (5–17)	<0.001 *

^#^ Chi^2^-test. * *p* =< 0.05.

## Data Availability

Data availability on request.
